# Understanding the Acceptability of Broadly Neutralizing Antibodies for HIV Prevention Among At-Risk Populations and Feasibility Considerations for Product Introduction in India: Protocol for a Qualitative Study

**DOI:** 10.2196/47700

**Published:** 2024-02-07

**Authors:** Joyeeta Mukherjee, Shruta Rawat, Saif ul Hadi, Pritha Aggarwal, Venkatesan Chakrapani, Pratyasha Rath, Pallavi Manchi, Srikrishnan Aylur, Shelly Malhotra, Margaret Keane, Alok Gangaramany

**Affiliations:** 1 International AIDS Vaccine Initiative Gurugram India; 2 The Humsafar Trust Mumbai India; 3 Centre for Sexuality and Health Research And Policy Chennai India; 4 The Final Mile Mumbai India; 5 Yeshwant Rao Gaitonde Centre for AIDS Research and Education Chennai India; 6 International AIDS Vaccine Initiative New York, NY United States

**Keywords:** HIV, key populations, acceptability, feasibility, product attributes, end-user preferences, broadly neutralizing antibodies, simulated behavioral experiments, qualitative study

## Abstract

**Background:**

Acceptability and preference research play a crucial role in the design, evaluation, and implementation of any new prevention product in any geographical setting. They also play a critical role in the development of clinical guidelines and policies. A wide range of acceptability studies have been conducted in diverse general and key populations for various new HIV prevention products worldwide. As clinical development strategies are being developed for clinical studies of broadly neutralizing antibodies (bNAbs) as potential HIV prevention products, appropriately tailoring them to address the type of HIV epidemic at hand would be critical for efficient uptake within in-country public health systems and decrease adoption and adherence challenges. Accomplishing this will require comprehensive acceptability and feasibility studies to inform multisectoral efforts that increase access to these products and national policies supportive of access to health care for those in most need. Thus, it is both opportune and important to undertake focused efforts toward informing product development strategies.

**Objective:**

This study aims to understand preferences for product attributes and key behavioral factors influencing adoption and uptake of bNAb prevention products among end-users including female sex workers, men who have sex with men, transgender women, people who inject drugs, and adolescent girls and young women in India and understand the key health system and programmatic perspectives toward the introduction of bNAb prevention products from health service providers and policy makers in India.

**Methods:**

A multisite study will be conducted in Delhi, Mumbai, and Chennai to capture the differences in perspectives among diverse end-users and key informants across the country. The study will use a multimethods design using focus group discussions, in-depth interviews, simulated behavioral experiments, and key informant interviews. A total of 30 focus group discussions, 45 in-depth interviews, 15 simulated behavioral experiments sessions, and 15 key informant interviews will be conducted across 3 sites.

**Results:**

The data collected and analyzed will enable insights on which specific product attributes matter the most to the populations and why some attributes are less preferred; contextual drivers of preferences and choices at individual, interpersonal, social, and structural levels; and relative positioning of bNAb products among other potential HIV prevention products. Insights from the health service providers and policy makers will provide a critical understanding of the need perception of the potential product in the existing product landscape and what additional efforts and resources are required for potential introduction, delivery, and uptake of the bNAb products in the Indian context.

**Conclusions:**

Insights generated from the abovementioned objectives will represent perspectives of populations of interest across geographies in India, will provide an overview of the acceptability of bNAb products and the feasibility of their introduction in this region, and will inform product development strategies.

**International Registered Report Identifier (IRRID):**

DERR1-10.2196/47700

## Introduction

### HIV Prevention Product Landscape

HIV/AIDS has been one of the most devastating epidemics in human history. Since the official recognition of *AIDS* in the early 1980s, a total of 77 million people have been infected globally and approximately half of them have succumbed to the disease. Although effective and affordable antiretroviral (ARV) treatment has transformed HIV from a *death sentence* to a chronic, manageable disease, people with HIV have shorter life expectancy than those without the virus. New infections continue to fuel the epidemic while disproportionately affecting low- and middle-income countries. India has the third largest HIV epidemic in the world, with 2.1 million people currently living with HIV, and the sixth highest incidence with 88,000 new infections per year [1]. In addition, the high variability and ever-changing face of the virus along with the persistent marginalization and stigmatization of populations most at risk remain critical challenges in controlling the spread of HIV infection [2].

Current HIV prevention strategies include a range of behavioral and biomedical interventions, including behavior change campaigns, promotion of consistent use of male and female condoms, use of clean needles and syringes, opioid substitution therapy (eg, buprenorphine), voluntary male medical circumcision, and biomedical tools such as the use of ARV medication as preexposure prophylaxis (PrEP), and the treatment of people living with HIV to reduce viral load to undetectable levels and prevent onward transmission (treatment as prevention). There are several new HIV prevention products in the pipeline that have the potential to reduce the spread of HIV infection, including next-generation ARVs, especially long-acting ARVs [3], intravaginal (dapivirine) ring [4], preventive vaccines, and broadly neutralizing monoclonal antibodies (bNAbs) [5-11]. Multiple studies have highlighted that a single product will not be preferred by all end- users under all circumstances [12,13] because there is a constant evolution in the life journeys, social contexts, and product attributes. Thus, different products may be preferred by end-users at different times. Introduction of a new product does not necessarily mean that the importance or relevance of existing products has been replaced. It is important to identify the unique value of novel interventions and understand end-user perspectives to highlight their value addition to the current prevention landscape. There is a need to create a toolbox and choice sets that will cater to the unique needs of end-users [14]. bNAbs are antibodies that can potentially be used to fight against multiple strains of HIV and are poised to have advantages over other preventive drugs such as the ability to target and act against a broad spectrum of viral strains, longer half-life and hence less frequent dosage, no risk of development of resistance to ARVs used for treatment, and comparatively safe with rare cases of adverse side effects [11,15-19]. More than 200 different bNAbs targeting HIV have been isolated to date, varying significantly in their coverage of global HIV isolates (breadth) as well as the amount required to neutralize the virus (potency). bNAbs have been proven to be safe for humans and can work effectively in preventing HIV infection. The antibody-mediated prevention trial found that an antibody called VRC01 could prevent HIV infection with specific HIV strains when administered every 8 weeks to adults [20]. In addition, combinations of bNAbs engineered with extended half-lives and increased potency are also being developed and tested in trials to ensure lower dosage and lesser frequency that would make the end product accessible and affordable. The World Health Organization (WHO) has also recently published the preferred product characteristics for monoclonal antibodies for HIV prevention [21], and it highlights that based on the needs of various target populations, the antibodies should have longer lasting duration of protection, have minimal side effects, and be delivered as injections, among others. In India, most of the abovementioned prevention options have not yet been rolled out as a part of the national policy, and hence, there is a need to understand the acceptability for bNAbs and their preference vis-à-vis other potential options.

### Acceptability and Feasibility Studies for bNAbs

#### Value of Acceptability and Feasibility Studies

Acceptability and preference research play a crucial role in the design, evaluation, and implementation of any new product in any geographical setting. They also play a critical role in the development of clinical guidelines and policies [22]. A wide range of acceptability studies have been conducted in diverse general and key populations for HIV prevention products including women, adolescent boys and girls, men who have sex with men (MSM), female sex workers (FSWs), people who inject drugs (PWID), and transgender women (TGW) [23-26]. These studies have focused on prevention products such as oral and injectable PrEP, vaginal and rectal microbicides, vaginal rings, and future HIV vaccines [22,26-29]. The main drivers of prevention product adoption and uptake include product effectiveness, cost, absence of side effects, and multipurpose protection against sexually transmitted diseases and pregnancy [30]. Acceptability studies have shown that emotional and intuitive decision-making for a product choice is also driven by factors such as sexual satisfaction, dimensions of trust, self-efficacy, and sociocultural environments [26,31]. There is evidence for differential preference for prevention products among different populations. For example, in South Africa, FSWs preferred injectable products over oral PrEP and microbicide gel, adolescents preferred a potential HIV vaccine and expressed dislike for a vaginal ring, and MSM preferred rectal microbicides [24,25]. Relative importance of specific product attributes also varied across target populations. For example, the route of PrEP administration was found to be the most important attribute for prospective end-users in Peru, Ukraine, India, and Botswana; FSWs in Kenya; and young women in South Africa [32], whereas the PrEP dispensing site was the most important attribute for participants from Uganda and MSM in South Africa and the second most important attribute for FSWs in Ukraine [32].

There are several standard qualitative methods for assessing the acceptability of an intervention for the target population and setting. Two common methods are focus group discussions (FGDs) and in-depth interviews (IDIs). Moderated FGDs allow for understanding of group dynamics including similarities and differences and allow to probe further on particular topics as they come up in the discussion. This type of interaction generally results in a deeper understanding of the forces in a community that may impede or facilitate the implementation of effective interventions. IDIs are conducted in a one-on-one setting with members of the target communities. They allow for deeper probing to understand more details, have nuanced understanding of sensitive topics, and have an opportunity to monitor changes in tone and body language, which is also an important factor in eliciting key responses.

For the successful development and adoption of any product, it is critical that perspectives from all service providers including health care professionals and community “gatekeepers” are taken into account in the development process [33,34]. Thus, there is a need to gather evidence from diverse populations and multitude of related stakeholders across geographies in India and focus on understanding their perspectives on potential new products such as bNAbs and preferences of different product attributes. These are often conducted through key informant interviews (KIIs).

In India, acceptability and feasibility studies have been conducted for various microbicides and PrEP [35-42]. These studies have been scattered over time and have been restricted to a few geographical locations and key populations. Thus, there is a need to gather evidence from diverse populations and multitude of related stakeholders across geographies in India and also focus on perspectives with respect to the potential new product—bNAbs.

#### Behavioral Science for Contextualized Understanding of End-User Decision-Making

Apart from the aforementioned methods, there are other qualitative research methods that are necessary to complement some of the emerging insights from the potential end-users. It has often been observed that studies conducted to explore preferences for hypothetical products or willingness to participate in hypothetical trials have shown differences in the stated preferences and intentions versus the eventual decisions and observed behavior of end-users [43-45]. In Kenya, an observational cohort study reported a 90% willingness to participate in future trials; however, during actual trial recruitment, only 30% were willing to actually enroll [44]. In a real-time scenario, end-users may be presented with multiple contextual trade-offs as well as nonconscious factors that may influence their decision-making, and hence, stated versus revealed preferences may be different [45]. It has also been reported that hypothetical choice experiments with end-users provide relatively satisfactory predictive results for positive choices, but they are less effective for predicting negative choices [43]. Thus, to premeditate and elicit the “say-do” gap, one of the useful methods deployed by behavioral scientists is to identify and analyze shortcuts known as heuristics in action, which take off the cognitive load of decision-making and help anticipate and explain potential avenues for gaps between the stated intention and action [46,47].

The cognitive process marking the final behaviors exhibited and decisions made by prospective end-users may be a mix of conscious and nonconscious factors and, hence, cannot always be brought out through in-person discussions alone. Therefore, the application of behavioral science in a simulated context has been found to be extremely beneficial for deciphering the key drivers of decisions and behaviors. In such a situation, the participants in a study do not just have to talk about their past or future decisions but have to actively make a decision in real time in a simulated environment. This provides researchers with a better understanding of the decision pathways of prospective end-users as well as potential aspects of say-do gaps to inform the design and development of products. Toward this, Ethnolab is a behavioral research methodology that often uses a Conundrum game [48] to deploy scenarios based on the preliminary qualitative research findings in a simulated environment and observe user choices in real time. The method has been used in several sociobehavioral and acceptability studies for health programs and interventions, including voluntary male medical circumcision decision-making in Zimbabwe and Zambia [48]; understanding HIV prevention for adolescent girls and young women (AGYW) in South Africa [49]; and estimating the impact of nonconscious drivers of human behavior among pregnant women and mothers of infants as well as with frontline workers on reproductive, maternal, newborn, and child health outcomes in Uttar Pradesh [50].

### Population of Interest

As noted previously, India has the third largest HIV epidemic in the world, with 2.1 million people living with HIV, and the sixth highest number of new infections at 88,000 per year [2]. Therefore, ensuring the acceptability and feasibility of any new potential HIV prevention product in the regional Indian context and needs is critical. The HIV epidemic in India is concentrated among key populations such as FSWs, MSM, PWID, and TGW. The average national prevalences from the government studies range from 1% to 6% (PWID: 6.3%, TGW: 3.1%, MSM: 2.7%, and FSWs: 1.6%) with high interstate variations [51], and access to HIV prevention and treatment services for these populations remain hindered by sociobehavioral and structural barriers including multilayered stigma, discrimination, and marginalization [52,53]. In addition to epidemiological and behavioral diversity across regions and key populations, the rapidly evolving nature of the epidemic as well as changes in risk behaviors owing to the rise in the use of new communication technologies also necessitate systematic and structured inquiry into the health system and end-user contexts and preferences impacting the eventual adoption and uptake of new HIV prevention products such as bNAbs. In addition, although there has been a steady decline in HIV prevalence in India over the last decade, the decline among men has been significantly more rapid than that among women [54]. Similarly, significant gender-based gaps persist in knowledge on HIV prevention among young people (aged 15-24 years); although 75% of the women had heard of HIV (as compared with 89% of men), only 43% of the women in the country knew where to get tested for HIV, and of them, only 14% were ever tested and received results [54]. In addition, the prevalence of intimate partner violence among women of reproductive age (15-49 years) remains high at 22% [54]. Thus, in addition to key populations, it also becomes important to understand the perspectives of AGYW to cover diverse viewpoints and preferences for HIV prevention products, specifically bNAbs.

### Study Objectives

The study objectives are as follows:

1. To understand preferences for product attributes and key behavioral factors influencing the adoption and uptake of bNAb prevention products among end-users including FSW, MSM, TGW, PWID, and AGYW populations in India
2. To understand key health system and programmatic perspectives toward the introduction of bNAb prevention products from health service providers and policy makers in India

## Methods

### Study Population and Sites

The study populations include potential end-user populations such as FSWs, MSM, TGW, PWID, and AGYW and key stakeholders such as health service providers (including frontline health care workers, community-based organization [CBO] or nongovernmental organization [NGO] representatives, and lead community representatives) and policy makers (including national, state, and district level officials or program managers).

The multisite study will be conducted in Delhi, Mumbai, and Chennai regions to ensure coverage of perceptions from North, West, and South India, including differences in perspectives among diverse end-users and stakeholders. These sites also have very high HIV prevalence among the study populations [51].

### Conceptual Framework

#### Conceptual Framework for Acceptability

**Figure 1:**
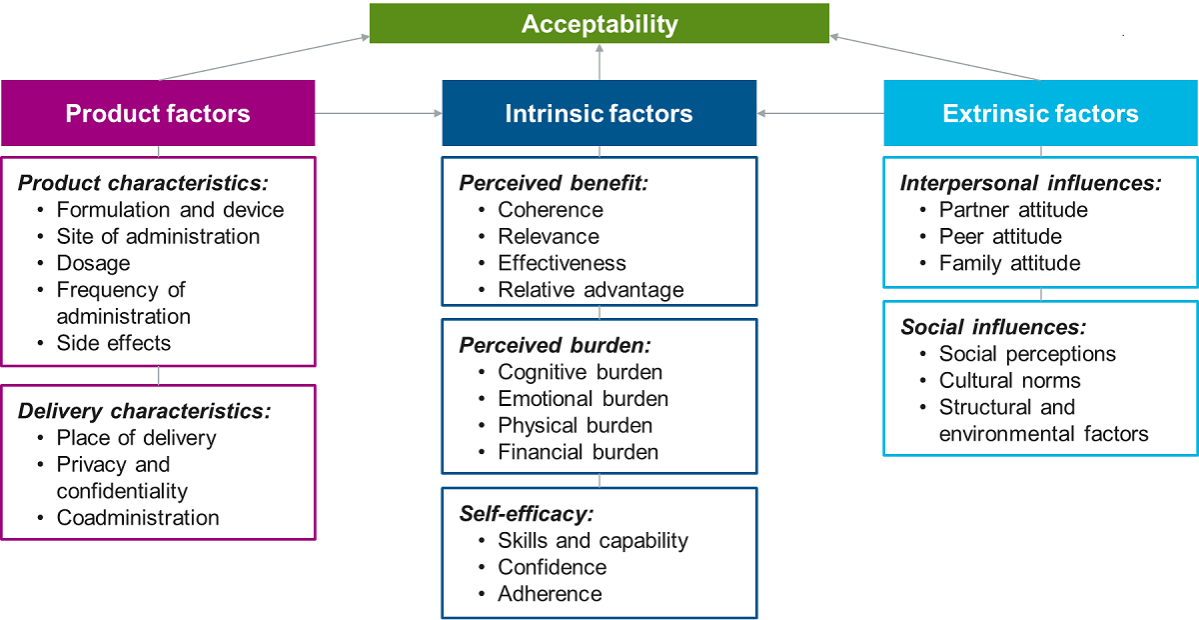
Conceptual framework for acceptability (adapted from the Theoretical Framework of Acceptability and the conceptual framework for product acceptability).

Acceptability may be defined as the extent to which people delivering or receiving a health care intervention consider it to be appropriate based on anticipated or experienced cognitive and emotional responses to the intervention [55]. Several theoretical frameworks are available for understanding acceptability of new HIV prevention products or technologies [41,42,55-57] at the individual and community levels. A recent systematic review proposed a model of acceptability of health interventions, the Theoretical Framework of Acceptability (TFA) [55]. According to TFA, intention to use a health intervention is determined by seven factors: (1) perceived effectiveness, (2) intervention coherence, (3) affective attitudes, (4) burden, (5) ethicality, (6) self-efficacy, and (7) opportunity costs. It was chosen to adapt the TFA to guide exploration of acceptability of bNAbs in this study as it can readily incorporate individual- and community-level influences on attitudes and behavior. The study design was also informed by the conceptual framework developed by Mensch et al [56] to incorporate the various factors operating at individual, interpersonal, community, and structural levels in influencing a person’s product-related attitudes and perceptions. In addition to adopting aspects from the social-ecological model, the framework emphasizes the role of actual product-related factors, including product attributes, which also play a role in defining an individual’s preferences [56]. Thus, guided by these frameworks, an adapted conceptual framework was used to design this study. In this adaptation, the constructs were embedded within three domains (Figure 1) [55,56]:

1. Product factors: those related to the potential product characteristics (formulation, device, site of administration, dosage, frequency of administration, and side effects) and delivery characteristics (place of delivery, privacy and confidentiality, and coadministration)
2. Intrinsic factors: those related to a person’s individual traits and not influenced by external factors. These include a person’s those related to a person’s individual traits and not influenced by external factors. These include a person’s *perceived benefit* of the product (coherence, relevance, effectiveness as well as relative positioning, and advantage of the product among other options), *perceived burden* in using the product (cognitive burden—complexity of dosage and time; emotional burden—fear, anxiety, and discreteness; physical burden—pain, side effects, and impact on sex; and financial burden—cost, travel, and opportunity loss), and *self-efficacy* (skills and capability, confidence, and adherence)
3. Extrinsic factors: those related to external influences including *interpersonal influences* (peer, partner, and family attitude) and *social/structural influences* (social perceptions—stigma and discrimination, cultural norms, and structural or environmental factors).

#### Conceptual Framework for Feasibility

**Figure 2:**
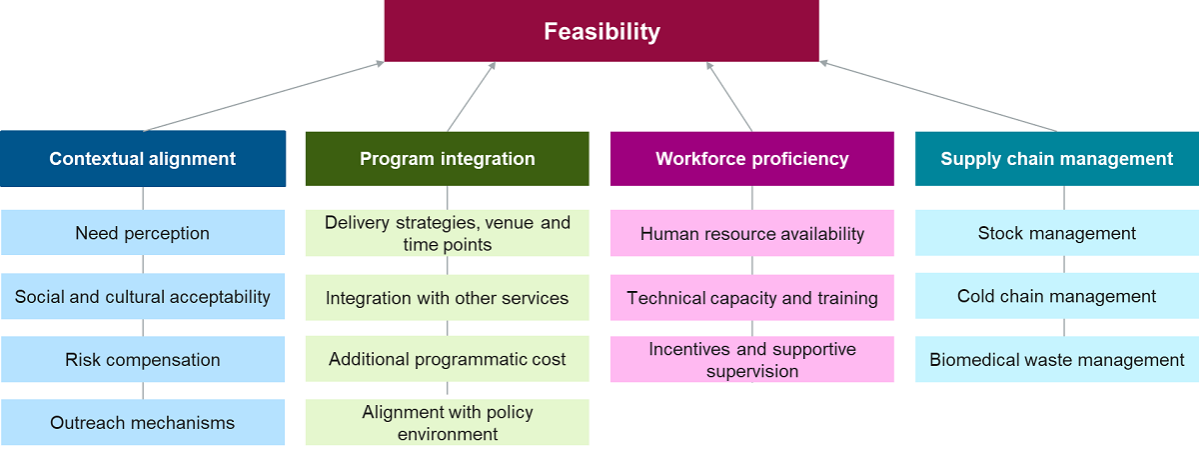
Framework for the assessment of feasibility of introduction of potential broadly neutralizing antibody products for HIV prevention (adapted from a study by World Health Organization).

There are multiple factors at the health systems and programmatic levels that service providers such as frontline health care providers, policy makers, and program managers at national, state, and district levels would consider as key issues that are needed for introduction and uptake of bNAbs as HIV prevention products in the future. Thus, complementing the study’s conceptual framework for acceptability, which will be used among the end-users, there is a need to have a conceptual framework to assess the feasibility. For this purpose, the framework shown in Figure 2 [58] was adapted from the WHO Framework on Health Systems, which was also used by the WHO for reviewing the impact of new vaccine introduction on immunization and health systems. The broad themes include the following:

1. Contextual alignment: This will enable an understanding of the need perception of the current product, its social and cultural acceptability, the outreach mechanisms that will be needed, and whether there is any risk compensation because of the introduction of this new product.
2. Program integration: This is toward understanding delivery strategies, venues, and time points; possibility of integration with already existing services; additional programmatic costs required; and alignment with the existing policy environment in India.
3. Health care workforce: This will explore the availability of adequate human resources—their distribution and workload, existing technical capacity and additional training needs, and incentives and supportive supervision required to successfully implement the introduction of a potential new product.
4. Integrated supply chain: This will be toward assessing the health systems’ readiness in terms of robust stock management, cold chain availability, and biomedical waste management.

### Study Design

#### Data Collection Methods

##### Overview

To understand preferences with regard to product attributes and key behavioral factors influencing the adoption and uptake of bNAb prevention products, this study will use a multimethods design [59] to provide a holistic approach based on complementarity and corroboration [60] by including the following qualitative methods:

- FGDs
- IDIs
- Simulated behavioral experiments (SBE)
- KIIs.

##### Data Collection Through FGDs

FGDs will be conducted among various groups such as FSWs, MSM, PWID, TGW, and AGYW. These discussions aim to understand the preferences and aspirations of prospective end-users regarding characteristics of HIV prevention products. In addition, the research seeks to uncover the key factors influencing their decision-making process including influential individuals and other contextual factors. Furthermore, the aim is to correlate these insights with what motivates or dissuades them from favoring specific HIV prevention options over others. This will be conducted in a small group of 7 to 8 participants. The end-users will also be asked about specific delivery preferences that would guide their adoption of and adherence to novel HIV biomedical prevention tools. FGDs allow the members of the group to collectively brainstorm on the issues, choices, drivers, and solutions, sometimes called a “synergistic group effect” [61]. This also allows for assessing participant behavior in a group setting wherein not only the individual respondent’s views are deliberated on, but the voices of the community as a whole are also accounted for.

##### Data Collection Through IDIs

IDIs will be conducted with additional participants from all the target populations through one-to-one discussions and will cover aspects similar to the FGDs. However, these will provide an opportunity to gather deeper insights as the interviewer can probe more, note the changes in respondent’s tone and body language, and also have views that are not influenced by group dynamics or social desirability [62].

##### Data Collection Through SBEs

SBEs will be conducted in small groups of 5 to 6 end-users to delve deeper into the causative factors of decision-making and potential avenues for intention-action gaps by using interdisciplinary concepts from behavioral economics and decision science. SBEs will use the insights generated from the FGDs and IDIs to construct a range of decision scenarios that end-users could face in deciding their preferences for a potential prevention product. SBEs will then use a game-based research technique (Ethnolab) to understand the context, emotions, and decision levers that influence decision-making based on these scenarios. Ethnolab is designed to minimize the impact of biases in decision-making, such as fear of value judgment, social desirability, and expectation of higher self-control in the future, which make respondents claim behaviors that may not play out in real life, thereby giving rise to “say-do” gaps [48]. Ethnolab would allow participants to not only discuss preferred attributes but also make real-time decisions revealing their preferences through multiple test scenarios. One of the critical objectives is to decipher the patterns of preference exhibited by different respondents across divergent contexts. Therefore, Ethnolab will help to identify the following:

- Past experiences that have led to decision-making criteria, values, norms, or beliefs that influence health behaviors and product preference
- Present goals around health management, relationship management, and life management and how they influence attribute selection
- Factors that could lead to preference reversal.

##### Data Collection Through KIIs

In addition, to better understand the environmental, market, programmatic, and policy contexts at the national, state, and local levels, along with decision-making factors and pathways informing HIV policy, KIIs will be conducted with policy makers and health service providers to understand their perspectives on drivers for the adoption and uptake of health innovations. Efforts will also be directed toward seeking their input on implementation and access issues as well as health systems challenges on non–cold chain versus cold chain product requirements and other parameters related to effective delivery and adoption of biomedical interventions to prevent HIV.

#### Sample Selection

The following sample selection criteria will be followed for the abovementioned methods.

##### End-Users

The inclusion criteria for the various target groups are:

- Inclusion criteria for MSM, TGW, PWID, and FSWs in the FGDs and IDIs were as follows: Aged at least 18 years, self-identifies as MSM, TGW, PWID, or FSW, and willing to provide informed consent.
- Inclusion criteria for AGYW in the FGD and IDIs were as follows: Aged 18 to 24 years and willing to provide informed consent. According to the Joint United Nations Programme on HIV and AIDS definition, AGYW fall into the age group of 15 to 24 years [63]. However, given the ethical considerations with respect to minor adolescent girls (15-17 y) in the Indian context and keeping in mind their relatively lower exposure to HIV prevention services, it was decided that for the purpose of this study, only adolescent girls aged 18 to 19 years and adult young women aged 20 to 24 years will be included. Efforts will be made during data collection and data analysis to ensure that perspectives from participants aged 18 to 19 years and those aged 20 to 24 years are disaggregated to bring out the unique highlights from these populations.
- For FGDs, diverse typologies and subgroups of MSM (
  kothis [feminine/primarily receptive role], panthis [masculine and primarily insertive role]
  , double-deckers or versatile, gay, and bisexual men), FSWs (brothel or bar based, street based, internet or social media based, and home based), TGW (gharana based and nongharana and those in sex work and those who are not), PWID (who use different types of injectable drugs), and AGYW across the age group (adolescents aged 18-19 years and young women aged 20-24 years) will be recruited.
- To explore diverse perspectives, maximum variation purposive sampling [64] will be used to recruit individuals from diverse backgrounds such as engaging in sex work; being from lower and middle class; and being single, living with male or female partners, heterosexually married, and representative of male and female PWID groups.

##### Key Informants

For KIIs, the inclusion criteria will be as follows:

- Key informants should be aged >18 years, ≥5 years of experience as a community leader or ≥3 years of working experience with population of interest or as a part of the HIV program, and willing to provide informed consent.
- Key informants will include information-rich individuals who have worked with key populations or have some experience of working with AGYW or in the field of HIV. These may include health service providers (physicians, counselors, nurses, CBO or NGO representatives, and community leaders) and policy makers (program managers and district or state or national level officials).

#### Sample Size

A total of 30 FGDs (2 FGDs per end-user group per site; for 5 end-user groups across 3 sites) and 45 IDIs (3 IDIs per end-user group per site; for 5 end-user groups across 3 sites) will be conducted in Delhi, Mumbai, and Chennai to investigate factors at individual, interpersonal, and sociostructural levels that facilitate or impede the acceptability of bNAbs among susceptible communities in India.

The SBE will mirror the sampling frame and the broad respondent profiles from the FGDs and IDIs. Keeping in mind that Ethnolab elicits the most useful insights in small and intimate settings, the sample would include 6 participants for each end-user group (distinct and different from the participants in the FGD or IDIs) across each site. Thus, the total sample size would be 90. Each instance of Ethnolab will have 6 respondents, and a total of 20 Ethnolab-based SBEs will be conducted across all 3 sites.

In addition, 5 KIIs will be conducted with health care service providers and policy makers in each region, amounting to a total of 15 KIIs. Thus, the total sample size of the qualitative component of the study is expected to be approximately 300 (FGD: 30×8=240, IDI=45, and KII=15).

#### Implementing Partners

The implementing partners for the study are YR Gaitonde Centre for AIDS Research and Education (YRGCARE), Centre for Sexuality and Health Research and Policy (C-SHaRP), and The Humsafar Trust (HST), who will be responsible for conducting the FGDs, IDIs, and KIIs. The designing of the Ethnolab game–based data collection method will be done by Final Mile and IAVI. Final Mile, in working with other study partners (YRGCARE, C-SHaRP, and HST), will be responsible for the implementation and conduct of the SBE sessions across all study sites.

### Data Collection

#### Overview

Data from FGDs, IDIs, and KIIs will be collected using semistructured interview topic guides. Trained research staff will conduct the FGDs, IDIs, and KIIs. The research staff will receive additional training specific to the content of this study and research ethics. SBEs will be conducted by partner consultants and will also include a round of initial meetings with the other study clinical research center partners to ensure alignment with the findings from the FGDs and IDIs.

#### Lines of Enquiry for FGDs and IDIs

For the FGDs and IDIs among end-users, the following broad areas will be covered in the lines of inquiry:

1. Preference elicitation questions: Under this process, the group will be asked about individual product attributes and their preference for different features under that attribute. The process will aim to understand which attributes matter most to the populations and the preferences for different subgroups under that population to gain an overall community-level perspective. Efforts will also be made to understand the reasons for these preferred options. This section would also cover the individual, interpersonal, and social concerns that might drive end-user decision-making.
2. Relative desirability of predetermined product profiles (attribute bundles): As a next step, participants within each focus group will be presented with 4 product profiles comprising 5 to 6 attributes in different combinations (bundles) through a mixture of visual methods and information, education, and communication tools. These are modeled after various HIV prevention products, including the current bNAb product under investigation. Participants will be requested to choose their “most/least preferred” profiles, rank the remaining 2, and explain the reasons behind their choice. In addition to enabling a deeper understanding of preferences and trade-offs, this would also aid in understanding the most critical attributes informing decision-making on product use among prospective end-users.

The topic guide will be translated into native languages (Hindi, Tamil, Telugu, or Marathi), back-translated into English, and then revised in the original language. All discussions will be audio-recorded, transcribed, and translated. They will be deidentified before proceeding for further data analysis. Each FGD and each IDI will span over 60 to 90 minutes.

#### Data Collection Process for SBE

The SBE sessions will consist of three parts:

1. *Ethnolab Conundrum game*: The Conundrum game will expose participants to multiple scenarios, each ending with a decision conundrum having different possible outcomes. Each scenario will simulate the real-world context of participants by representing a protagonist of the same age, gender, and socioeconomic level in a relevant health-related situation. The scenarios will be constructed using preliminary insights generated from the FGDs and IDIs, with decision points informed by heuristics and behavior science principles. The research is gamified: participants will be asked to respond quickly in real time and choose the option they think will coincide with most respondents in the group and will win points if it does. The game will thus generate responses representative of mental models, emotions, and biases rather than deliberate and rational analysis [48]. Preference reversal will be tested through tweaks in the context simulation and the framing of attributes.
2. *Ideal product profile exercise*: The participants will go through a series of prompts to design the ideal prevention product suited to their needs. This activity will be an adapted version of the product profile ranking exercise used in the first round of qualitative research. It will be designed to give participants an expanded choice set to clearly articulate their trade-offs and allow for tracing certain preference reversal patterns. This will enable an overall high engagement and easy and detailed discussion on product attributes.
3. *Group conversations*: Once the games are over, participants will be split into multiple groups of dyads and triads for group conversations (where scenarios and reactions are still fresh in their minds) regarding the experience, decisions, and preferences in the game [50]. The conversations on the scenarios provide opportunities for improved contextualization of the responses, understanding emotions and other causative factors behind preferences, and tracing decision pathways.

The data generated from the games, along with the ensuing conversations, will help elicit behavioral insights and identify intrinsic drivers and extrinsic factors influencing the preferences and decisions of prospective end-users. The Ethnolab play will last for approximately 60 minutes, and the subsequent group discussions will last for 45 minutes. The overall activity will not exceed 2 hours. The session will involve engaging games to maintain a playful environment, and adequate breaks will also be incorporated to ensure that the time of engagement does not become burdensome for the participants.

#### Lines of Enquiry for KIIs

Semistructured interview guides will consist of 10 to 12 open-ended questions and scripted probes. The interview guides will be translated into native languages (Hindi, Tamil, or Marathi), back-translated into English, and then revised in the original language. Key informants for each study population will be asked questions for the need perception of a prevention product, preference and importance of product attributes, social and cultural acceptability, risk compensation, health systems readiness, and alignment with the policy environment in India. Each KII will be conducted for approximately 90 minutes.

### Data Analysis and Validation

#### Analysis

The qualitative approach will use triangulation to strengthen and ensure the accuracy of data [65]. According to Denzin and Lincoln [66], triangulation is a process in which several methods are used in the study and might be used in four basic ways: (1) data triangulation, (2) methods triangulation, (3) theory triangulation, and (4) researcher triangulation. In this study, data triangulation, methods triangulation, and researcher triangulation will be used. All transcripts will be redacted (to remove any personally identifying information that might inadvertently have been recorded) and uploaded into NVivo (Lumivero) or Dedoose (University of California). First, a line-by-line review of the transcripts will be performed, and first-level codes will be identified to create a common a priori codebook (based on the adapted conceptual or theoretical framework and topic guides). All codes will be then entered and tagged to associated chunks of text. Texts corresponding to each of the first-level codes will be coded by at least 2 independent analysts and reviewed by senior investigators. Explanatory or theoretical or etic (researcher generated) codes will be arrived at using a constant comparative method [67].

The SBE results will be analyzed using the cognitive and emotional appraisal framework [68] as a base. Appraisal theories are componential theories that view an emotional episode as involving changes in a number of organismic subsystems and components. The components could include an appraisal, a motivational, a somatic, and a motor component. The framework will be used to generate the codebook and will then feed into the 4 major categories of context, emotions, mental models, and decision levers. This will be done along with the ranking of preferences drawn from the experiential game data. The SBEs will be analyzed to gain an in-depth understanding of (1) preference construction and (2) preference reversal.

#### Validation

Data source triangulation and researcher triangulation will enhance the reliability and trustworthiness of the findings [69]. Thus, study implementation and data analysis will also incorporate researcher reflexivity and researcher triangulation by maintaining “quicknotes” (a brief summary of every interview or focus group by the interviewers or moderators that will be shared with the analysts and investigators for feedback), writing memos, and engaging in peer debriefing to maintain ongoing awareness of our social location and how it may influence the research process and interpretation. A maximum of 2 interviewers or moderators will be used for every population of interest to ensure that there is minimum bias in the way questions are asked or follow-up probes are used.

#### Data Security for Storage and Transmission

Names or other personal identifiers will not be collected from the participants. Only the investigators and key research staff at the participating research sites will have access to the transcripts or translated text and digital recordings. As soon as an interview is completed, the audio file will be transferred from the digital recorder to password-protected computers. Subsequently, the file will be deleted from the digital recorder. The transcriptionist and translator will sign a confidentiality pledge stating that they will not reveal any information from the interviews to anyone else. The digital copies of transcripts and translated text will also be stored on password-protected computers.

Only this study’s research staff at the participating research site will have access to the audio files, transcripts, and translated text. All digital recordings will be redacted, and any personal identifying information will be removed. Digital recordings will be deleted after 1 year (the 1-year delay is to ensure that there are no gaps in the transcripts). Redacted transcripts will be maintained for 2 years and then destroyed. Informed consent forms will be separately stored and will include signs, initials, or “X” marks rather than names. Unique identification codes will be assigned to all individual records, including digital recordings and transcripts. Hard copies of the data and related documents will be stored in a secure location in locked cabinets in the offices of study and will be accessed only by the research staff.

### Ethical Considerations

#### Institutional Review Board Review and Approvals

The study was presented to the institutional review boards (IRBs) of all 3 implementing partners (HST, C-SHaRP, and YRGCARE), and all comments received were adequately addressed in the protocol. The approved protocol (HST-IRB-50-01/2021; C-SHaRP/0007327/220; YRGCARE-359) will be implemented for data collection and analysis.

#### Informed Consent Process

Participants for IDIs, KIIs, FGDs, and SBEs will be presented with an information sheet that outlines the scope of the study and a consent form that provides options to sign or put initials or put an “X” mark. This approach has been accepted by the IRB for other previous studies. Given the potentially low literacy levels of some participants, the research assistants will offer to read and explain the information sheet and consent form to the participants. As part of the informed consent process, all potential participants will be informed that their decision to participate or not in interviews or games will not affect the services they currently receive or may receive in the future from their respective community agencies.

#### Compensation

Participants in the FGDs, IDIs, and SBEs will receive an honorarium of INR 500 (US $6.25) for participating in the study. This amount is based on input from the community advisory boards of the study partners in India. This covers the costs incurred for roundtrip transportation. If the focus group or IDI participants elect to withdraw from the study during data collection, they will still receive compensation. Key informants will receive INR 1500 (US $18.75) (wherever applicable), and this is proposed from previous learning wherein key informants reported loss of wages owing to their participation and suggested that an honorarium should be instituted for KIIs to minimize such experiences. All interviews will be conducted in person, and honorariums will be in cash.

#### Privacy and Confidentiality Protection of Participants and Do-No-Harm Measures

##### Overview

Being a qualitative study, with no biological samples collection, there is no medical risk to a research participant of this study. The study also does not pose any financial risk to the participants. However, participants’ confidentiality could be compromised through engagement in the study, and participants might feel uncomfortable answering questions about sexual behaviors. Recognizing these possibilities, some mitigation measures that will be adopted are as follows:

1. Unique identity number and no names: No participant names will be collected. Participants will be asked to mark only their initials or put an “X” mark on the consent form. Participants will be identified only by a unique numerical identifier. Individuals will be instructed not to use their surnames but to use a nickname or pseudonym. In addition, all transcripts will be anonymized so that any identifying information that is inadvertently mentioned will be removed from the written transcript.
2. Training for research interviewers or FGD moderators: Interviewers or moderators will be well trained in research ethics and interviewing techniques. They will be trained to be sensitive to the needs of the participants to stop, rest, skip particular questions, or discontinue their involvement in the study at any time. Interviewers will be clearly instructed that participants are allowed not to answer any question and continue with the interview or to stop the interview at any time. In addition, research interviewers and field research staff will be trained in research ethics and confidentiality and will all be required to sign confidentiality agreements.
3. Explicit information on potential social risks to participants of focus groups: For focus groups, social risks include unwanted disclosure of one’s sexual orientation or sexual behaviors and facing possible stigma or discrimination if a member of the larger community discovered the participant’s orientation or drug use or sex work status. Furthermore, confidentiality will be maintained by holding interviews and focus groups at discreet locations that many of the participants already frequently visit (eg, local CBO or NGO office). In addition, facilitators and interviewers will be trained in research ethics and there is already a list of transcribers or translators who possess sufficient experience in transcribing research interviews. They are also well versed in the significance of upholding confidentiality and anonymity.
4. Transcription and translation of FGD, IDI, KII, and behavioral experiments: Professional transcribers will be hired for the transcription or translation of audio-recorded interviews (FGDs, IDIs, KIIs, and behavioral experiment discussions). There already exists a list of transcribers or translators with adequate experience in transcribing research interviews and who know about the importance of maintaining confidentiality and anonymity. They (new or experienced) will be oriented about the importance of maintaining the confidentiality of the audio files and transcripts and will be asked to keep all the research information confidential by not discussing or sharing the content of the interviews in any form or format with anyone other than the designated research contact persons. The transcriber or translator will be asked to sign a confidentiality pledge before transcription or translation.

##### Do-No-Harm Measures

In addition to the mentioned measures, all study staff will be trained in guidelines and policies related to human participant research ethics to ensure no unintended harm to the participants. This will be applicable for all participants, keeping in mind their vulnerabilities, but especially noteworthy for engagement with adolescent populations. The study teams will adhere to the National Youth Policy and also be trained in the guidelines stated by the Rashtriya Kishore Swasthya Karyakram [70].

##### Plan for Reporting Unanticipated Problems or Study Deviations

The study investigators will report unanticipated problems and study deviations to the local IRB. Such events will also be communicated to International AIDS Vaccine Initiative for further review or escalation, if required. Minor problems and protocol deviations, if any, (which pose no risk to participants or others) will be reported in the annual protocol continuing review.

##### COVID-19–Related Guidelines

All study data collection–related activities will be conducted by adhering to the state or national guidelines in view of COVID-19.

#### Possible Benefits to Participants

There are no direct benefits to participants for taking part in this study. However, the participants will be better informed about the various HIV prevention options currently available across the globe and the options under development. The study has the potential to benefit the society and the participant’s communities if the data collected are found effective and are adopted in routine service delivery or for further informing bNAb research.

## Results

The study is being implemented across 3 sites in India among 5 different populations and key informants. The data collection will be followed by data analysis workshops to enable standardization of analysis across all sites and partners. Extensive analysis based on the analytical frameworks will lead to the expected outcomes of the study.

## Discussion

### Principal Findings

The study will provide critical insights into factors driving the end-user decision-making with regard to prevention product choices and assess the acceptability of bNAbs in the Indian context. It will also help understand the health system and programmatic factors that would influence product introduction and uptake among target populations in India. The specific anticipated outcomes from the study can be highlighted as follows:

1. *Product preferences*: Insights from the FGDs, IDIs, and SBEs will aid in identifying specific product attributes that matter the most to the end-users and why some attributes have less preference. Within each attribute, it will be possible to identify the options that the respondents feel are more significant to their individual context. Insights will also be generated on end-user preferences that are stable across all contexts and preferences that are dynamic and may vary with changing circumstances. Finally, it will also be possible to outline the acceptability and unique value proposition of bNAb products in the Indian context.
2. *Behavioral factors*: Another outcome of the study will be identification of the most relevant target populations in India for HIV bNAb products and user segmentation of the target population based on key behavioral determinants, including an interplay between user profiles and preferences. The study will also help understand the contextual drivers of preferences and choices at individual, interpersonal, social, and structural levels and aid in delineating the key decision levers and pathways that lead to a preference or reversal of the same. These decision levers could be understood in the form of norms, values, goals, mental models, and emotions and would be critical to decipher to understand acceptability of a product. In addition, often, the stated preferences are different from the observed choices when they are simulated through real-life situations. Thus, the gamified components will also provide a chance to highlight the say-do gap to inform product development.
3. *Health systems and programmatic readiness*: Insights from the health service providers and policy makers will provide a critical understanding of the needs perception of the potential product in the existing product landscape, understanding of the cultural and social acceptability, and risk compensation. Their views will also provide critical insights into the additional efforts and resources required for potential introduction, delivery, and implementation of bNAb products in the Indian context. This will include program integration perspectives and other structural factors such as workforce availability and supply chain integration. The study will also enable an understanding of the existing policy landscape and alignment of the potential product with the same toward informing the feasibility of introduction.

Insights generated from all the abovementioned outcome domains will represent perspectives of populations of interest across geographies in India and will be collated into management presentations to provide an overview of the acceptability and feasibility of bNAb products in this region toward informing product development strategies.

### Limitations of the Study

We anticipate some limitations. First, being a qualitative study, an inherent limitation of the study is that the findings may not be generalizable (in a statistical sense) as purposive sampling, a nonprobability sampling widely used in qualitative research, was used. However, we will try to ensure diversity when recruiting potential participants from various study subgroups, that is, by using maximum variation or diversity sampling, a type of purposive sampling. Such a strategy, along with a detailed description of the settings and participant characteristics, will increase the chances of the transferability of the findings to similar contexts and populations. Second, the study is being implemented by 3 study partners in different settings. Although all interviewers used standardized data collection tools and underwent common trainings, personal differences in interviewing styles may result in varied elicitation of responses from end-users. We will try to reduce this bias by having periodic debriefing sessions with the research team, especially data collectors, which will help the team to reflect on how one’s values and beliefs might influence the way we ask questions in the interviews or FGDs or in arriving at inferences when analyzing the data [71].

### Conclusions

With the evolution in the HIV prevention research field and the expansion of the prevention toolbox, end-users have a wide spectrum of choices. It is important for product developers to understand the drivers of these choices to clearly articulate the unique value proposition of each product to define their positioning in the choice spectrum. It is also critical to generate timely evidence on target populations, facilitators and barriers of use, relative importance of products, and communication needs to facilitate, plan, and enable policy decisions for the introduction of newer products. Thus, this study will aid in generating insights that will be critical for product developers and policy makers.

## Abbreviations

AGYW: adolescent girls and young women
ARV: antiretroviral
bNAb: broadly neutralizing antibody
CBO: community-based organization
C-SHaRP: Centre for Sexuality and Health Research and Policy
FGD: focus group discussion
FSW: female sex worker
HST: Humsafar Trust
IDI: in-depth interview
IRB: institutional review board
KII: key informant interview
MSM: men who have sex with men
NGO: nongovernmental organization
PrEP: preexposure prophylaxis
PWID: people who inject drugs
SBE: simulated behavioral experiment
TFA: Theoretical Framework of Acceptability
TGW: transgender women
WHO: World Health Organization
YRGCARE: YR Gaitonde Centre for AIDS Research and Education

## Data Availability

The data sets generated during the study will be used to analyze the final findings from the study. The analyzed data sets will be available in the main manuscript that will be published highlighting the findings from this study. For data sharing, national policies applicable to Indian research data [72] will be adhered to.

## References

[1] Global HIV and AIDS fact sheet. Joint United Nations Programme on HIV/Aids.

[2] Seyler L, Lacor P, Allard SD (2018). Current challenges in the treatment of HIV. Pol Arch Intern Med.

[3] Cohen J (2020). Long-acting injectable drug prevents HIV infections. Science.

[4] Cairns G (2020). The vaginal ring against HIV: regulatory approval for a new prevention choice for women. aidsmap.

[5] Walker LM, Phogat SK, Chan-Hui PY, Wagner D, Phung P, Goss JL, Wrin T, Simek MD, Fling S, Mitcham JL, Lehrman JK, Priddy FH, Olsen OA, Frey SM, Hammond PW, Kaminsky S, Zamb T, Moyle M, Koff WC, Poignard P, Burton DR, Protocol G Principal Investigators (2009). Broad and potent neutralizing antibodies from an African donor reveal a new HIV-1 vaccine target. Science.

[6] Wu X, Yang ZY, Li Y, Hogerkorp CM, Schief WR, Seaman MS, Zhou T, Schmidt SD, Wu L, Xu L, Longo NS, McKee K, O'Dell S, Louder MK, Wycuff DL, Feng Y, Nason M, Doria-Rose N, Connors M, Kwong PD, Roederer M, Wyatt RT, Nabel GJ, Mascola JR (2010). Rational design of envelope identifies broadly neutralizing human monoclonal antibodies to HIV-1. Science.

[7] Huang J, Ofek G, Laub L, Louder MK, Doria-Rose NA, Longo NS, Imamichi H, Bailer RT, Chakrabarti B, Sharma SK, Alam SM, Wang T, Yang Y, Zhang B, Migueles SA, Wyatt R, Haynes BF, Kwong PD, Mascola JR, Connors M (2012). Broad and potent neutralization of HIV-1 by a gp41-specific human antibody. Nature.

[8] Shingai M, Nishimura Y, Klein F, Mouquet H, Donau OK, Plishka R, Buckler-White A, Seaman M, Piatak Jr M, Lifson JD, Dimitrov DS, Nussenzweig MC, Martin MA (2013). Antibody-mediated immunotherapy of macaques chronically infected with SHIV suppresses viraemia. Nature.

[9] Sok D, van Gils MJ, Pauthner M, Julien JP, Saye-Francisco KL, Hsueh J, Briney B, Lee JH, Le KM, Lee PS, Hua Y, Seaman MS, Moore JP, Ward AB, Wilson IA, Sanders RW, Burton DR (2014). Recombinant HIV envelope trimer selects for quaternary-dependent antibodies targeting the trimer apex. Proc Natl Acad Sci U S A.

[10] Mendoza P, Gruell H, Nogueira L, Pai JA, Butler AL, Millard K, Lehmann C, Suárez I, Oliveira TY, Lorenzi JC, Cohen YZ, Wyen C, Kümmerle T, Karagounis T, Lu CL, Handl L, Unson-O'Brien C, Patel R, Ruping C, Schlotz M, Witmer-Pack M, Shimeliovich I, Kremer G, Thomas E, Seaton KE, Horowitz J, West Jr AP, Bjorkman PJ, Tomaras GD, Gulick RM, Pfeifer N, Fätkenheuer G, Seaman MS, Klein F, Caskey M, Nussenzweig MC (2018). Combination therapy with anti-HIV-1 antibodies maintains viral suppression. Nature.

[11] Schommers P, Gruell H, Abernathy ME, Tran MK, Dingens AS, Gristick HB, Barnes CO, Schoofs T, Schlotz M, Vanshylla K, Kreer C, Weiland D, Holtick U, Scheid C, Valter MM, van Gils MJ, Sanders RW, Vehreschild JJ, Cornely OA, Lehmann C, Fätkenheuer G, Seaman MS, Bloom JD, Bjorkman PJ, Klein F (2020). Restriction of HIV-1 escape by a highly broad and potent neutralizing antibody. Cell.

[12] Chang LW, Serwadda D, Quinn TC, Wawer MJ, Gray RH, Reynolds SJ (2013). Combination implementation for HIV prevention: moving from clinical trial evidence to population-level effects. Lancet Infect Dis.

[13] Laher F, Salami T, Hornschuh S, Makhale LM, Khunwane M, Andrasik MP, Gray GE, Van Tieu H, Dietrich JJ (2020). Willingness to use HIV prevention methods among vaccine efficacy trial participants in Soweto, South Africa: discretion is important. BMC Public Health.

[14] Godfrey-Faussett P, Frescura L, Abdool Karim Q, Clayton M, Ghys PD, (on behalf of the 2025 prevention targets working group) (2022). HIV prevention for the next decade: Appropriate, person-centred, prioritised, effective, combination prevention. PLoS Med.

[15] Horwitz JA, Halper-Stromberg A, Mouquet H, Gitlin AD, Tretiakova A, Eisenreich TR, Malbec M, Gravemann S, Billerbeck E, Dorner M, Büning H, Schwartz O, Knops E, Kaiser R, Seaman MS, Wilson JM, Rice CM, Ploss A, Bjorkman PJ, Klein F, Nussenzweig MC (2013). HIV-1 suppression and durable control by combining single broadly neutralizing antibodies and antiretroviral drugs in humanized mice. Proc Natl Acad Sci U S A.

[16] Barouch DH, Whitney JB, Moldt B, Klein F, Oliveira TY, Liu J, Stephenson KE, Chang H, Shekhar K, Gupta S, Nkolola JP, Seaman MS, Smith KM, Borducchi EN, Cabral C, Smith JY, Blackmore S, Sanisetty S, Perry JR, Beck M, Lewis MG, Rinaldi W, Chakraborty AK, Poignard P, Nussenzweig MC, Burton DR (2013). Therapeutic efficacy of potent neutralizing HIV-1-specific monoclonal antibodies in SHIV-infected rhesus monkeys. Nature.

[17] Scheid JF, Horwitz JA, Bar-On Y, Kreider EF, Lu CL, Lorenzi JC, Feldmann A, Braunschweig M, Nogueira L, Oliveira T, Shimeliovich I, Patel R, Burke L, Cohen YZ, Hadrigan S, Settler A, Witmer-Pack M, West Jr AP, Juelg B, Keler T, Hawthorne T, Zingman B, Gulick RM, Pfeifer N, Learn GH, Seaman MS, Bjorkman PJ, Klein F, Schlesinger SJ, Walker BD, Hahn BH, Nussenzweig MC, Caskey M (2016). HIV-1 antibody 3BNC117 suppresses viral rebound in humans during treatment interruption. Nature.

[18] Caskey M, Schoofs T, Gruell H, Settler A, Karagounis T, Kreider EF, Murrell B, Pfeifer N, Nogueira L, Oliveira TY, Learn GH, Cohen YZ, Lehmann C, Gillor D, Shimeliovich I, Unson-O'Brien C, Weiland D, Robles A, Kümmerle T, Wyen C, Levin R, Witmer-Pack M, Eren K, Ignacio C, Kiss S, West Jr AP, Mouquet H, Zingman BS, Gulick RM, Keler T, Bjorkman PJ, Seaman MS, Hahn BH, Fätkenheuer G, Schlesinger SJ, Nussenzweig MC, Klein F (2017). Antibody 10-1074 suppresses viremia in HIV-1-infected individuals. Nat Med.

[19] Bar KJ, Sneller MC, Harrison LJ, Justement JS, Overton ET, Petrone ME, Salantes DB, Seamon CA, Scheinfeld B, Kwan RW, Learn GH, Proschan MA, Kreider EF, Blazkova J, Bardsley M, Refsland EW, Messer M, Clarridge KE, Tustin NB, Madden PJ, Oden K, O'Dell SJ, Jarocki B, Shiakolas AR, Tressler RL, Doria-Rose NA, Bailer RT, Ledgerwood JE, Capparelli EV, Lynch RM, Graham BS, Moir S, Koup RA, Mascola JR, Hoxie JA, Fauci AS, Tebas P, Chun TW (2016). Effect of HIV antibody VRC01 on viral rebound after treatment interruption. N Engl J Med.

[20] Corey L, Gilbert PB, Juraska M, Montefiori DC, Morris L, Karuna ST, Edupuganti S, Mgodi NM, deCamp AC, Rudnicki E, Huang Y, Gonzales P, Cabello R, Orrell C, Lama JR, Laher F, Lazarus EM, Sanchez J, Frank I, Hinojosa J, Sobieszczyk ME, Marshall KE, Mukwekwerere PG, Makhema J, Baden LR, Mullins JI, Williamson C, Hural J, McElrath MJ, Bentley C, Takuva S, Gomez Lorenzo MM, Burns DN, Espy N, Randhawa AK, Kochar N, Piwowar-Manning E, Donnell DJ, Sista N, Andrew P, Kublin JG, Gray G, Ledgerwood JE, Mascola JR, Cohen MS (2021). Two randomized trials of neutralizing antibodies to prevent HIV-1 acquisition. N Engl J Med.

[21] WHO preferred product characteristics for monoclonal antibodies for HIV prevention. World Health Organization.

[22] Griffin JB, Ridgeway K, Montgomery E, Torjesen K, Clark R, Peterson J, Baggaley R, van der Straten A (2019). Vaginal ring acceptability and related preferences among women in low- and middle-income countries: a systematic review and narrative synthesis. PLoS One.

[23] Kennedy C, Fonner V, Kennedy C, Fonner V (2016). Pre-exposure prophylaxis for people who inject drugs: a systematic review. Consolidated Guidelines on HIV Prevention, Diagnosis, Treatment and Care for Key Populations – 2016 Update.

[24] Quaife M, Eakle R, Cabrera Escobar MA, Vickerman P, Kilbourne-Brook M, Mvundura M, Delany-Moretlwe S, Terris-Prestholt F (2018). Divergent preferences for HIV prevention: a discrete choice experiment for multipurpose HIV prevention products in South Africa. Med Decis Making.

[25] Atujuna M, Newman PA, Wallace M, Eluhu M, Rubincam C, Brown B, Bekker LG (2018). Contexts of vulnerability and the acceptability of new biomedical HIV prevention technologies among key populations in South Africa: a qualitative study. PLoS One.

[26] Bauermeister JA, Downs JS, Krakower DS (2020). PrEP product acceptability and dual process decision-making among men who have sex with men. Curr HIV/AIDS Rep.

[27] Koechlin FM, Fonner VA, Dalglish SL, O'Reilly KR, Baggaley R, Grant RM, Rodolph M, Hodges-Mameletzis I, Kennedy CE (2017). Values and preferences on the use of oral pre-exposure prophylaxis (PrEP) for HIV prevention among multiple populations: a systematic review of the literature. AIDS Behav.

[28] Patel SK, Rohan LC (2017). On-demand microbicide products: design matters. Drug Deliv Transl Res.

[29] Eakle R, Weatherburn P, Bourne A (2019). Understanding user perspectives of and preferences for oral PrEP for HIV prevention in the context of intervention scale-up: a synthesis of evidence from sub-Saharan Africa. J Int AIDS Soc.

[30] Humphrey JM, Naanyu V, MacDonald KR, Wools-Kaloustian K, Zimet GD (2019). Stated-preference research in HIV: a scoping review. PLoS One.

[31] Eakle R, Bourne A, Jarrett C, Stadler J, Larson H (2017). Motivations and barriers to uptake and use of female-initiated, biomedical HIV prevention products in sub-Saharan Africa: an adapted meta-ethnography. BMC Public Health.

[32] Eisingerich AB, Wheelock A, Gomez GB, Garnett GP, Dybul MR, Piot PK (2012). Attitudes and acceptance of oral and parenteral HIV pre-exposure prophylaxis among potential user groups: a multinational study. PLoS One.

[33] Krakower DS, Mayer KH (2016). The role of healthcare providers in the roll out of pre-exposure prophylaxis. Curr Opin HIV AIDS.

[34] Venter WD (2018). Pre-exposure prophylaxis: the delivery challenge. Front Public Health.

[35] Joglekar NS, Joshi SN, Deshpande SS, Parkhe AN, Katti UR, Mehendale SM (2010). Acceptability and adherence: findings from a Phase II study of a candidate vaginal microbicide, 'Praneem polyherbal tablet', in Pune, India. Trans R Soc Trop Med Hyg.

[36] Tolley EE, Tsui S, Mehendale S, Weaver MA, Kohli R (2012). Predicting product adherence in a topical microbicide safety trial in Pune, India. AIDS Behav.

[37] Mehendale S, Deshpande S, Kohli R, Tsui S, Tolley E (2012). Acceptability of coitally-associated versus daily use of 1% tenofovir vaginal gel among women in Pune, India. Int Health.

[38] Prabhughate A, Sarna A, Brady M (2014). Tenofovir gel for HIV prevention for women: perspectives of key opinion leaders from India. Health Policy Technol.

[39] Chandhiok N, Joshi SN, Gangakhedkar R (2014). Acceptability of oral and topical HIV chemoprophylaxis in India: implications for at-risk women and men who have sex with men. Sex Health.

[40] Chakrapani V, Newman PA, Shunmugam M, Mengle S, Varghese J, Nelson R, Bharat S (2015). Acceptability of HIV pre-exposure prophylaxis (PrEP) and implementation challenges among men who have sex with men in India: a qualitative investigation. AIDS Patient Care STDS.

[41] Chakrapani V, Newman PA, Shunmugam M, Mengle S, Nelson R, Rubincam C, Kumar P (2017). "Like holding an umbrella before it rains": acceptability of future rectal microbicides among men who have sex with men in India-a modified technology acceptance model. Qual Health Res.

[42] Chakrapani V, Shunmugam M, Rawat S, Baruah D, Nelson R, Newman PA (2020). Acceptability of HIV pre-exposure prophylaxis among transgender women in India: a qualitative investigation. AIDS Patient Care STDS.

[43] Lambooij MS, Harmsen IA, Veldwijk J, de Melker H, Mollema L, van Weert YW, de Wit GA (2015). Consistency between stated and revealed preferences: a discrete choice experiment and a behavioural experiment on vaccination behaviour compared. BMC Med Res Methodol.

[44] Nyasani DK, Mutua GN, Sajabi RM, Ng'ang'a JW, Gachie JN, Maina AM, Lusike LL, Anzala AO, Price MA, Manyonyi GO (2018). Reported willingness to participate in a hypothetical HIV vaccine trial and its translation to actual participation among healthy adults-experience from Kenya. PLoS One.

[45] Strauss M, George G, Mantell JE, Romo ML, Mwai E, Nyaga EN, Odhiambo JO, Govender K, Kelvin EA (2018). Stated and revealed preferences for HIV testing: can oral self-testing help to increase uptake amongst truck drivers in Kenya?. BMC Public Health.

[46] Tversky A, Kahneman D (1974). Judgment under uncertainty: heuristics and biases. Science.

[47] Kanhemann D (2011). Thinking Fast and Slow.

[48] Sgaier SK, Sharma S, Eletskaya M, Prasad R, Mugurungi O, Tambatamba B, Ncube G, Xaba S, Nanga A, Gumede-Moyo S, Kretschmer S (2017). Attitudes and decision-making about early-infant versus early-adolescent male circumcision: demand-side insights for sustainable HIV prevention strategies in Zambia and Zimbabwe. PLoS One.

[49] Gomez A, Malone S, Prasad R, Gangaramany A, Croucamp Y, Mulhausen J, Noble-Campbell P, Balakrishnan D (2019). Understanding HIV prevention in high-risk adolescent girls and young women in two South African provinces. S Afr Health Rev.

[50] Engl E, Kretschmer S, Jain M, Sharma S, Prasad R, Ramesh BM, Shetye M, Tandon S, Kumar S, Barrios Wilson T, Sgaier SK (2019). Categorizing and assessing comprehensive drivers of provider behavior for optimizing quality of health care. PLoS One.

[51] (2017). HIV Sentinel Surveillance (HSS): technical brief. National AIDS Control Organization (NACO).

[52] Beattie TS, Bhattacharjee P, Suresh M, Isac S, Ramesh BM, Moses S (2012). Personal, interpersonal and structural challenges to accessing HIV testing, treatment and care services among female sex workers, men who have sex with men and transgenders in Karnataka state, South India. J Epidemiol Community Health.

[53] Chakrapani V, Velayudham J, Shunmugam M, Newman PA, Dubrow R (2014). Barriers to antiretroviral treatment access for injecting drug users living with HIV in Chennai, South India. AIDS Care.

[54] (2017). National family health survey (2015-16). Ministry of Health and Family Welfare.

[55] Sekhon M, Cartwright M, Francis JJ (2017). Acceptability of healthcare interventions: an overview of reviews and development of a theoretical framework. BMC Health Serv Res.

[56] Mensch BS, van der Straten A, Katzen LL (2012). Acceptability in microbicide and PrEP trials. Current Opinion in HIV and AIDS.

[57] Campbell JI, Aturinda I, Mwesigwa E, Burns B, Santorino D, Haberer JE, Bangsberg DR, Holden RJ, Ware NC, Siedner MJ (2017). The Technology Acceptance Model for Resource-Limited Settings (TAM-RLS): a novel framework for mobile health interventions targeted to low-literacy end-users in resource-limited settings. AIDS Behav.

[58] WHO-framework on health systems. World Health Organization.

[59] Hunter A, Brewer JD, Hesse-Biber SN, Johnson RB (2015). Designing multi-method research. The Oxford Handbook of Multimethod and Mixed Methods Research Inquiry.

[60] Mukherjee N, Zabala A, Huge J, Nyumba TO, Adem Esmail B, Sutherland WJ (2018). Comparison of techniques for eliciting views and judgements in decision‐making. Methods Ecol Evol.

[61] Sussman S, Burton D, Dent CW, Stacy AW, Flay BR (2006). Use of focus groups in developing an adolescent tobacco use cessation program: collective norm effects. J Applied Social Pyschol.

[62] Moris A, Morris A (2015). The what and why of in-depth interviewing. A Practical Introduction to In-Depth Interviewing.

[63] HIV prevention among adolescent girls and young women. Joint United Nations Programme on HIV/Aids.

[64] Crossman A (2020). Understanding purposive sampling: an overview of the methods and its applications. ThoughtCo.

[65] Lincoln YS, Guba EG (1985). Naturalistic Inquiry.

[66] Denzin NK, Lincoln YS (2015). The Sage Handbook of Qualitative Research.

[67] Strauss A, Corbin J (1998). Basics of Qualitative Research: Techniques and Procedures for Developing Grounded Theory.

[68] Frijda NH, Mesquita B, Mascolo MF, Griffin S (1998). The analysis of emotions. What Develops in Emotional Development?.

[69] Pope C, Ziebland S, Mays N (2000). Qualitative research in health care. Analysing qualitative data. BMJ.

[70] Rashtriya Kishor Swasthya Karyakram (RKSK). National Health Mission.

[71] Rankl F, Johnson GA, Vindrola-Padros C (2021). Examining what we know in relation to how we know it: a team-based reflexivity model for rapid qualitative health research. Qual Health Res.

[72] Data Protection guideline of the National AIDS Control Organization. National AIDS Control Organization.

